# Cytogenetic profile of Acute Myeloid Leukemia and Acute Lymphoblastic Leukemia in Northern Pakistan

**DOI:** 10.12669/pjms.39.5.6405

**Published:** 2023

**Authors:** Maria Khan, Chaudhry Altaf, Hamid Saeed Malik, Mohammad Abdul Naeem

**Affiliations:** 1 Dr. Maria Khan, MBBS, FCPS Hematology. Consultant Hematologist, Armed Forces Institute of Cardiology, Rawalpindi, Pakistan; 2 Dr. Chaudhry Altaf Hussain, MBBS, FCPS Hematology. Consultant Hematologist, CMH Institute of Medical Sciences, Bahawalpur, Pakistan; 3 Dr. Hamid Saeed Malik, MBBS, FCPS Hematology. Consultant Hematologist, Armed Forces Institute of Pathology, Rawalpindi, Pakistan; 4 Dr. Mohammad Abdul Naeem, MBBS, MCPS, FCPS Hematology, PhD, FRCP. Consultant Hematologist, CMH Lahore Medical College, Lahore, Pakistan

**Keywords:** Cytogenetics, Acute myeloid leukemia (AML), Acute lymphoblastic leukemia (ALL), Metaphase

## Abstract

**Objective:**

To determine the frequencies of different cytogenetic abnormalities in patients of Acute Myeloid Leukemia and Acute Lymphoblastic Leukemia in Northern Pakistan.

**Methods:**

It was descriptive cross-sectional study conducted in Hematology Department of a Tertiary care referral institute from June 2015 to July 2017. All newly diagnosed cases of Acute Leukemia were analyzed. Cytogenetic analysis was performed on bone marrow aspirate samples using Giemsa-trypsin banding technique. Karyotypes were identified and interpreted according to ISCN criteria.

**Results:**

A total of 355 newly diagnosed patients of Acute Leukemia were analyzed. Out of these, 180 patients had AML and 175 had ALL. In Acute Myeloid Leukemia chromosomal abnormalities were detected in 28.2 % cases. Of these the common ones included t(8;21),t(15;17),+8, Inversion 16 and Monosomy 7. Other abnormalities included Complex karyotype, Down’s syndrome related AML, Hyperdiploidy, del 16q,-8,+Y and t(3p;17q)del 10. In Acute Lymphoblastic Leukemia chromosomal abnormalities were detected in 40% cases. Common ones included Hyperdiploidy, Tetraploidy and t(9;22). Other abnormalities included t(1;19) and t(2;8)t(8;14).

**Conclusion:**

Cytogenetically favorable abnormalities are commonest occurring chromosomal defects in both Acute Myeloid Leukemia and Acute Lymphoblastic Leukemia in Northern Pakistan, i.e., t(8;21) in AML and Hyperdiploidy in ALL.

## INTRODUCTION

Acute Myeloid Leukemia (AML) and Acute Lymphoblastic Leukemia (ALL) are heterogeneous groups of neoplastic disorders characterized by clonal expansion of hematopoietic blasts of myeloid and lymphoid lineages respectively.^1^,^2^ Initially organized systematically by French-American-British cooperative group in 1976, Acute leukemias were categorized by taking in to account morphological and cytochemical parameters. However, limited clinical relevance along with insufficiency in elaborating underlying biological nature of disease and treatment stratification have been major drawbacks of this system.^3^ Therefore, a paradigm shift from FAB was made in WHO classification 2001 of Acute Myeloid and Lymphoblastic Leukemias and its revision in 2008 and lately in 2016.

This WHO system has adopted not only a lower blasts percentage of 20%, but also integrated cytogenetic and molecular abnormalities with clinical, morphological, cytochemical and immunophenotypic parameters for establishing diagnosis.^4^,^5^ Many of the chromosomal abnormalities have been incorporated in this classification in the category of AML and ALL with recurrent cytogenetic abnormalities. These unique chromosomal aberrations are associated with distinct morphological and immunophenotypic properties of blasts and have important prognostic implications.^6^-^8^

In the recent years an improved outcome has been observed in the patients of acute leukemias with identification and characterization of cytogenetic aberrations. This has paved a way for understanding the underlying pathological pathways of the disease as well as their prognostic implications. Thus, this advancement is aiding the clinicians in structuring cytogenetic based risk stratification and treatment options.^9^-^11^

## METHODS

This study was a cross-section analysis conducted in Hematology department of Armed Forces Institute of Pathology, a tertiary care referral institute, from June 2015 to July 2017 after approval from institute’s ethical review board (Ethical Review Board Certificate Number IRB/17/309, Dated August 22, 2017). All patients diagnosed as Acute Myeloid Leukemia and Acute Lymphoblastic Leukemia of all ages and both genders were included in the study. Cases of Acute undifferentiated Leukemias, Acute Bi-phenotypic/mixed lineage Leukemias, relapsed cases and patients who had received chemotherapy were excluded.

Complete blood counts, Peripheral film, Bone marrow aspiration and Trephine biopsies were performed on all patients after informed and written consent. Samples were sent to the Institute’s Molecular and Cytogenetics departments for molecular and cytogenetic analyses. All cases were diagnosed according to WHOs classification with the incorporation of clinical data, morphology, cyto-chemistry and immunophenotyping by flow cytometry.^5^,^12^

Cytogenetic analysis was carried out using conventional Giemsa trypsin banding (G-banding) technique. Three ml of bone marrow aspirate samples were taken in sodium heparin tubes. All samples were cultured in RPMI 1640 medium for next 48 hours. This was followed by harvesting to obtain metaphases by first adding 1% colchicine followed by incubation, then centrifugation and finally addition of hypotonic solution of 1%KCl. Fixative (3:1 methanol to glacial acetic acid) was added and slides were made. Geimsa staining of the slides was done followed by their examination under microscope to look for presence of at least 20 metaphases which rendered the culture successful. Lastly, Giemsa trypsin banding was performed and slides were analyzed by Cytovision semi-automated image analysis and capture system.

Chromosomal abnormalities were identified and interpreted according to the International System for Human Cytogenetic Nomenclature (2009,2013) criteria. Data was entered and analyzed using SPSS version 20. Parameters for analysis included age, gender and types of cytogenetic abnormalities and results were expressed as frequencies. Mean and standard deviation were calculated for quantitative variables like age, white blood cell count. Effect modifiers like age and gender type were controlled by stratification. Post stratification chi-square test was applied with P value ≤ 0.05 taken significant.

## RESULTS

A total of 376 patients of acute leukemia were diagnosed during the study period. Out of these 190 had acute myeloid leukemia and 184 acute lymphoblastic leukemia, while other two cases were of mixed phenotypic acute leukemia. Successful cytogenetic analysis was carried out in 180 patients of AML (94.5%) and in ten cases (5.5%) no metaphase chromosome was yielded. In ALL successful cytogenetic analysis was carried out in 175 patients (94.9%) and in nine cases (5.1%) no metaphase chromosome was yielded.

### Acute Myeloid Leukemia:

**Fig.1:**
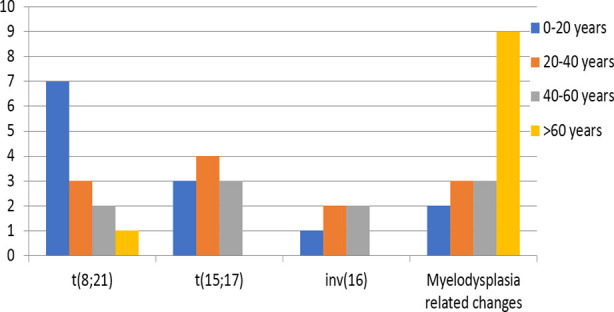
Age distribution pattern in Acute Myeloid Leukemia with cytogenetic abnormalities.

Out of 180 patients 108 were males and 72 were females with a male to female ratio of 1.5:1. Their median age was 47 years with age ranges between one year to 80 years. Chromosomal abnormalities were found in 51 (28.3%) patients while 129 (71.7%) patients had a normal karyotype. The frequencies of different cytogenetic abnormalities are given in Table-I. Data analysis revealed that recurrent cytogenetic abnormalities were the most commonly occurring chromosomal aberrations in AML seen in 29 (16.1%) cases followed by AML with myelodysplasia related changes found in 17 (9.4%) cases. Age distribution pattern in AML revealed that t(8;21) was more commonly observed in younger age group of 0-20 years, t(15;17) and inv(16) above 20 years of age and MDS in elderly population of more than 60 years. Characteristic features of AML and age distribution pattern in AML with cytogenetic abnormalities are shown in Table-I and Fig.1 respectively.

**Table-I:** Characteristics of Acute Myeloid Leukemia Patients

Characteristics	No. of patients (n)	Frequency (%)	Males	Females
***FAB Subtype***				
AML M0	2	1.1	1	1
AML M1	23	12.7	17	6
AML M2	91	50.5	52	39
AML M3	31	17.2	15	16
AML M4	18	10	11	7
AML M5a	3	1.6	3	0
AML M5b	1	0.5	1	0
AML M6	8	4.4	5	3
Pure Erythroid Leukemia	2	1.1	2	0
AML M7	1	0.5	1	0

Total	180	100	108	72

***Cytogenetic Results***				
Normal karyotype	129	71.66	84	45
Recurrent cytogenetic abnormalities	29	16.1	15	14
t(8;21)	14	7.8	8	6
t(15;17)	10	5.5	4	6
Inv(16)	5	2.8	3	2
Myelodysplasia related AML	17	9.36	7	10
Trisomy 8	8	4.4.	3	5
Monosomy 7	4	2.2	2	2
Complex karyotype	3	1.66	1	2
Del16q	1	0.55	-	1
-Y	1	0.55	1	-
Others	5	2.75	2	3
Hyperdiploidy	1	0.55	-	1
t(4q;21q)	1	0.55	1	-
t(12p;17q)	1	0.55.	-	1
Monosomy 8	1	0.55	1	-
t(3p ; 17q)	1	0.55	-	1

Total	180	100	108	72

### Acute Lymphoblastic Leukemia:

**Fig.2:**
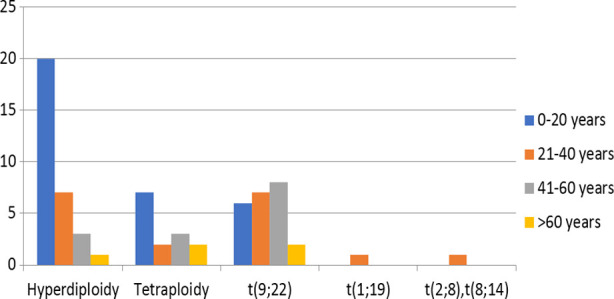
Age distribution pattern in Acute Lymphoblastic Leukemia with cytogenetic abnormalities.

There were 132 males and 43 females with a male to female ratio of 3:1. The median age at diagnosis was 34 years with age ranges between 6 months to 79 years. Among these, 164 patients were diagnosed as B-cell ALL while 11 patients had T-cell ALL. Chromosomal abnormalities were detected in 70 (40%) patients while 105 (60%) patients had normal karyotype. The frequencies of different cytogenetic abnormalities are given in Table-II. The age distribution pattern in ALL showed that hyperdiploidy and tetraploidy / near tetraploidy were more common in younger age group of 0-20 years while t(9;22) was more frequently observed in ages 20-60 years. Characteristic features of ALL and age distribution pattern in ALL with cytogenetic abnormalities are shown in Table-II and Fig.2 respectively.

**Table-II:** Characteristics of Acute Lymphoblastic Leukemia Patients

Characteristics	No. of patients (n)	Frequency (%)	Males	Females
***FAB Subtype***				
ALL-L1	101	57.7	72	29
ALL-L2	73	41.7	59	14
ALL-L3	1	0.57	1	0
Total	175	100	132	43
***Cytogenetic Results***				
Normal karyotype	105	60	85	20
l Hyperdiploidy	31	17.7	18	13
t(9;22)	23	13.1	16	7
Near tetraploidy	14	8	11	3
t(1;19)	1	0.57	1	-
t(2;8),t(8;14)	1	0.57	1	-

Total	175	100	132	43

## DISCUSSION

Valuable information can be retrieved from karyotyping in cases of hematological malignancies. The discovery of underlying chromosomal defects has revolutionized the management of acute leukemias by potentially developing and employing targeted therapeutic approach in addition to conventional chemotherapeutic options which may lead to improvised outcomes in a number of cases. Moreover, this information can play a pivotal role in the risk stratification and predicting long term survival and prognosis of the disease. Unfortunately, in Pakistan only a few advanced diagnostic centres are harbouring this facility and there are issues of logistics as well as affordability. Hence, we do not have detailed statistics of the cytogenetic defects in patients of acute myeloid leukemia and acute lymphoblastic leukemia in our population. To our knowledge, we have presented the most comprehensive cytogenetics data encompassing both AML and ALL from Northern Pakistan. Diverse ethnic representation was noted which included Northern Punjabis, Pashtoons, Kashmiris, Gilgit Baltistan ethnic origin and other smaller ethnic groups. Some important observations were made. In general, cytogenetic abnormalities were observed more frequently in Punjabis and Pashtoons. This may be due to their higher numbers in the study population.

In our study, cytogenetic abnormalities were found in 28.3% cases of AML, comparable with the statistics found in Malaysian study by Meng et al (30.4%).^13^ However this percentage is lower in comparison with frequency found in Chinese population by Cheng et al (58%).^14^ The difference can be due to ethnic, geographical and demographic differences along with the cytogenetic methodology used, as R- banding technique was adopted in Chinese study which has more sensitivity.

Recurrent cytogenetic abnormalities were the commonest occurring chromosomal defects in karyotypically abnormal cases of AML i.e., 16.1%. In this group, t(8;21) was the most frequent occurring defect seen in 7.8% cases, followed by t(15;17) found in 5.5% cases. These findings were in agreement with the frequency reported by Shaikh et al.^15^ with t(8;21) observed in 8.1% and t(15;17) in 5.5% of cases. These balanced translocations were more prevalent in the younger age group but were statistically insignificant. A local study conducted by Khalil et al on pediatric AML reported a higher incidence of t(8;21) in their patients which also supports the findings of our study of increased incidence of this chromosomal aberration in younger ages.^16^ Similar results were observed in the study conducted by Meng et al.^13^ These recurrent cytogenetic abnormalities were also found to be the commonest group in UK and China^8^,^14^,^17^ but t(15;17) was more prevalent than t(8;21) in these regions. This can be due to the fact that more patients diagnosed at AFIP were of FAB type AML-M2 than AML-M3. These cytogenetic abnormalities are known to harbor good prognostic significance.

Another important information derived from our data was high frequency of AML with myelodysplasia related changes in the elderly age group. This is again in agreement with the international data.^13^,^18^ Notable cytogenetic aberrations in this group include Trisomy 8 in agreement with the commonest abnormality reported by Li X et al. and Kadam Amare PS et al.^17^,^19^ which carries intermediate prognostic significance,^20^ Monosomy seven and Complex karyotyping, which are adverse prognostic indicators.^8^

Data analysis in ALL also revealed some noteworthy facts. Recurrent cytogenetic abnormalities were the sole defects identified in karyotypically abnormal cases, i.e., 40%. This percentage is comparable with Brazilian study carried out by Compos C et al. i.e., 45.8%.^21^ Hyperdiploidy was the most frequently occurring chromosomal aberration with majority of the cases involving childhood and adolescence. Hyperdiploidy is a prognostically favorable abnormality. This was followed by t(9;22) which was found to be more common in the adult age group. These findings are in accordance with international literature.^21^,^22^ A study representing the southern region of Pakistan also demonstrated increased frequency of Philadelphia chromosome in the adult ALL.^23^ Similar findings were observed in Sudanese population.^24^

An important observation was low percentage of t(1;19) and t(4;11) / MLL gene rearrangement, only one case was reported to have t(1;19) none had t(4;11) in our study. This fact has been strengthened by another local study which also ascertained the same finding.^22^ Also one patient diagnosed as ALL had t(2;8), t(8;14) characteristic of Burkitt’s lymphoma and at the time of diagnosis it had morphological features of FAB ALL-L2.

### Strength of the study:

Besides presenting the largest cytogenetic data in Pakistani population with AML and ALL, other strength of this study includes confirmation of diagnoses by expert morphologists followed by confirmed lineage specific characterization of all cases by flow cytometric immunophenotyping.So, our study on conventional cytogenetic analyses has revealed quite a number of non-random chromosomal abnormalities in Acute Leukemias. Many of these have relevant implications for their diagnosis and pathogenesis. In addition, this information also provides framework for treatment stratification in such cases.

We employed G-banding technique in our study; further sensitivity of cytogenetic analysis can be enhanced by using other more advanced methods like R-banding. Moreover, as cryptic rearrangements cannot be detected by conventional karyotyping and their suspicion mandate further evaluation by other tests like FISH, PCR etc.

Cytogenetic analysis is relatively new diagnostic modality in developing countries like Pakistan and concrete efforts are required for its progression by integrating conventional cytogenetics with multiplex / molecular analyses. This will eventually have a strong and positive impact on the outcome of patients with acute leukemias.

## CONCLUSION

We conclude that favorable cytogenetic abnormalities are commonest occurring chromosomal defects in both AML and ALL in our setup, i.e., t(8;21) and t(15;17) in AML and Hyperdiploidy in ALL.

### Author’s Contributions:

**MK:** Concept, study design, literature search, data acquisition and analysis, statistical analysis, manuscript preparation.

**CA:** Concept, study design, literature search, manuscript editing, review and approval

**HSM:** Concept, study design, manuscript review and approval

**MAN:** Literature search, manuscript approval

All authors are responsible and accountable for the accuracy and integrity of the work.
